# A Forward Look from the Society’s Present and Past Presidents: A Transcript from the 2023 ASTMH Annual Meeting

**DOI:** 10.4269/ajtmh.24-0146

**Published:** 2024-05-02

**Authors:** Daniel G. Bausch, Chandy C. John, James W. Kazura, N. Regina Rabinovich, David H. Walker

**Affiliations:** ^1^ FIND, Geneva, Switzerland;; ^2^ Indiana University, Indianapolis, Indiana;; ^3^ Case Western Reserve University, Cleveland, Ohio;; ^4^ Harvard TH Chan School of Public Health, Boston, Massachusetts;; ^5^ Department of Pathology, University of Texas Medical Branch, Galveston, Texas

**Dan Bausch:**

Good morning and thanks for coming. This is the ASTMH President's Address. I’m Dan Bausch, the current President. Some of you who attended last year’s meeting in Seattle would have heard my President’s Address on the nexus of tropical medicine and human rights. If you didn’t hear it, I encourage you to pull it up on YouTube. Having had my say last year, I thought that people might not have patience for another Dan Bausch soliloquy this year, and so thought we would try something a little different with this year’s Address.

I’ve asked some of my ASTMH Past-President colleagues to join me on stage for a discussion to offer, I hope, the wisdom of the ages. Let me first state that many Past-Presidents were interested and willing to participate, but with only 45 minutes for this session, having a group of 25 people was going to be difficult. Hence, we had to limit it to the six of us on the stage now. I nevertheless thank all the Past-Presidents for their support for this session.

Let me introduce the panel, starting with myself: As you know, I’ve been privileged to be your President for the last two years (2022–23). Then we have my immediate predecessor, Julie Jacobson (2021), Chandy John (2019), Regina Rabinovich (2018), David Walker (2013), and Jim Kazura (2012). So, we have representation stretching across at least the last decade of our evolving Society.

I’d like to separate this session into three broad categories. We’ll start with questions that students, trainees, and young investigators have sent to me in response to a request sent out a few months ago. Then I would like to get broader as we go; we’ll talk about the state of our Society from a broader but still internal perspective, and then finally go more global, getting the panels’ ideas on the world today, the Society’s place in it, and the challenges and opportunities that face us.

I have questions that will fill the whole time, but I hope we don’t have to use them because I would like this to be more of a discussion with the audience. This is your opportunity to ask questions of the ASTMH leadership! I’ll kick us off for a few minutes but, when you’re ready, please come up to the mic and enter into the conversation. Thanks very much and we’ll start:

First question, which is distilled from various trainees and young investigator submissions: What skill sets should the Society offer trainees beyond what they receive in their academic homes? Anyone want to take that on? Julie?

**Julie Jacobson:**

I think we have heard a pretty loud and clear cry for more voices to be heard more broadly in ongoing debates. So, I think the skill that is needed is really communication skills, writing op-eds and taking an active voice communicating the importance of science. Karen Goraleski, in her CEO role, has always had a communications and advocacy session here at the meeting, but I think there’s more skills in that area to be developed, and I think the Society could take an active role in that because it’s going to be so important moving forward. That’s just one example, I’m sure of many, that could be provided by the Society.

**James Kazura:**

I can add some comments to Julie’s points. It was a different era when I served as President of ASTMH (2012-2013) in the sense that the power of social media was in its nascence. Discussions in the previous few years were centered on issues such as changing the name of the Society and its journal from “tropical medicine” to “global health” given the former phrase’s association with colonialism. Clearly, the Society has changed in a positive way since then. As Julie indicates, the Society’s meeting and journal are more multidisciplinary, ethnically and geographically diverse, and include topics related not only to biological aspects of disease but also social justice and economic variables.

**Figure 1.**
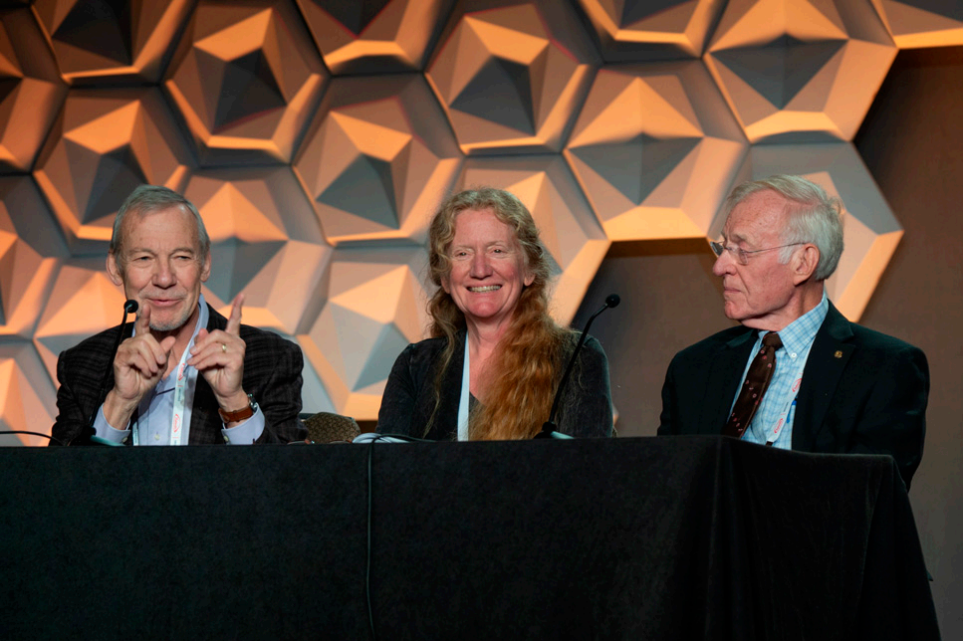
Past-Presidents (from left) Jim Kazura, Julie Jacobson, and David Walker.

**Chandy John:**

Just building on that, I think that the greatest strength of the Society is the ability to collaborate across disciplines. So, this isn’t a Society for any one particular thing only. There are smaller groups for that, but it’s a group that brings together all the different things in tropical medicine and global health, including basic science and translational work and clinical work, but also public health and bigger issues like climate change.

One of the things that has been really helpful for me is that I’ve built so many collaborations through the people I’ve met here, but maybe one thing the Society can do even better is teach you how to do that. We sort of do it on our own because you meet people or you go to talks and you think, “Oh, this is cool, let’s talk about this.” But how can we build upon that to leverage the greatest asset of this Society, which is its diversity of disciplines and working together?

**Regina Rabinovich:**

I think everyone is quite aware of the challenge that science and scientists have in being part of the voice of what science is real and what is fake news. And that takes courage because you are dealing with some very strong activist communities who feel that their version of good science or data is the right one. And for those like Peter Hotez and others that have spoken up, whether it’s anti-vaccine or Ivermectin with COVID, or any of the contentious issues that exist today, it’s scary, because you take a risk. You are a target, and you will get attacked on social media.

So, I think depending on your academic track, it is clear that the press can call you and ask, “What do you know about dengue in Bolivia and what’s happening with dengue?” Those are facts! The challenge is how to deal with fake news and take your communication skills to a different level, particularly on what topics you are willing to take risks in speaking to the press or the public directly. That’s not taught in academia. That’s something that we do have models within the Society for how to do and how to manage those risks, but we need to be brave.

**Dan Bausch:**

Thanks all. I’ll come to you, David, in just a minute, but first just one comment from me in relation to science and fake news: If you weren’t at the “Science Under Assault” session the other day with Peter Hotez, and also the session “A Scientist’s Cheat Sheet to Understanding Washington, DC” yesterday, these questions were addressed there in some very interesting ways. If you missed them, remember that part of your paid registration includes access to the recordings of the entire meeting. I don’t know exactly when they will come out, but you’ll soon be able to go back and catch what you’ve missed. I would definitely recommend these two sessions. Over to you, David.

**Figure 2.**
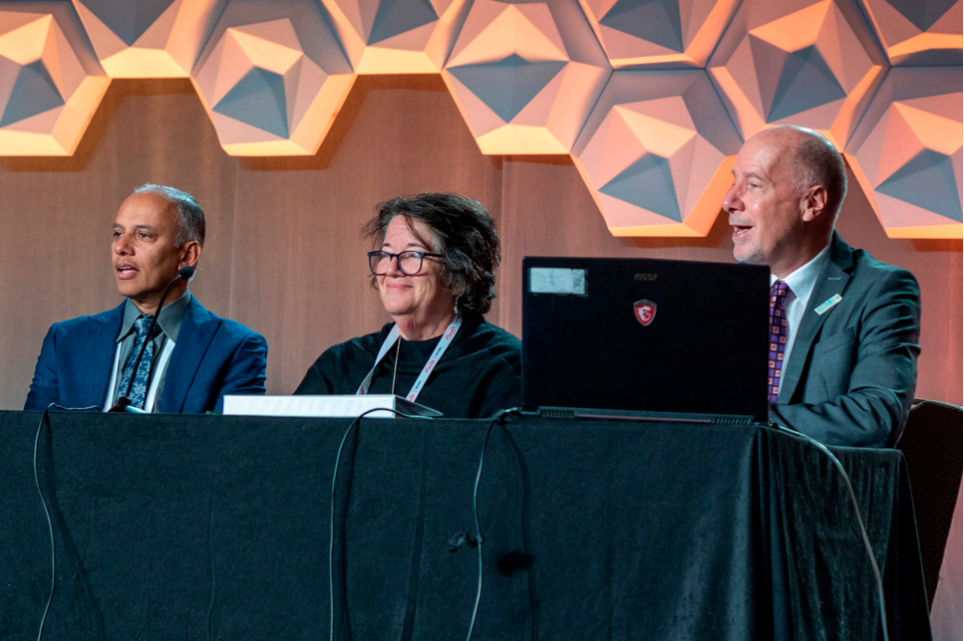
Past-Presidents (from left) Chandy John, Regina Rabinovich, and Dan Bausch.

**David Walker:**

One of the things that we offer and should be doing more of is mentorship. I think we need to, at this meeting, identify people that we can interact with in an ongoing basis, not just at a meeting, but throughout the year and develop a relationship to help to identify and create the next generation of leaders. This includes people from every country, not just people that are working in laboratories in the United States, and develop their ability to be leaders, to understand, to push the frontier of knowledge forward and to help them find the way to accomplish that.

**Dan Bausch:**

Thanks, David. Some years ago we tried to establish an ASTMH mentoring program. There were many mentees but far fewer volunteer mentors, and so it was hard to put together. But I do agree that it’s time for the Society to revisit this in some way. As you look around the room, there’s incredible potential of both younger and older people. Trying to somehow bring them together to capitalize on that experience and wisdom as we all grow and transition in our careers is a really important thing for us to do.

Another question from the trainees: How to reconcile competing demands of your training and the research that you’re doing in your lab and the advocacy required for implementation? We’re all about that these days, I think, recognizing that simply producing scientific evidence is not sufficient. But, especially for a younger person, how do you move towards that translational part of it into implementation to make sure your work in the lab has impact?

**Regina Rabinovich:**

Embedded in that is a critical decision: If you’ve had something coming out of your lab, which needs to become a product, needs to become an intervention, are you willing? Is what you aspire to, to be that driving force and leave the lab, leave what you were doing and begin really working to drive? We have excellent examples of people that grew up in ivory towers and are out in huge field trials and whose products we are seeing today.

Or do you want to advocate for it, and hand it off to others? Do you want to talk about what the needs are, what the next steps are? Figuring out who needs to listen to what you have come up with and help do that translation? Do you want to create linkages or do you want to figure out how to do it yourself? And then what is the pathway for that? They’re both choices, it’s fine, but that’s the critical first decision I think that needs to be made.

**Julie Jacobson:**

I think this is a really important point, and I think it is one of the things the Society can be a part of because we include so many cross-disciplinary areas. Sitting in the audience are program managers from programs in endemic countries. They understand the needs and the challenges that they’re facing and those voices need to be heard. Once you appreciate the challenges, you’re looking at the questions that you’re asking scientifically in your research and seeing how are those aligned with the needs of the field, and ensuring that you have those conversations. I think we should facilitate a lot more of those conversations as part of the Society.

There’s a meeting, the Coalition for Operational Research on NTD (COR-NTD) meeting, that happens around the annual ASTMH meeting where they focus on these issues. I think we can bring that kind of thinking into ASTMH as well and try and get some networking opportunities to bring together the many brilliant minds out there. ASTMH is just such an incredible collection of people, and if all of the interesting things that members are working on were aligned towards the key challenges that people are having in countries. Just imagine the power of that.

I think being intentional and asking the question is already a significant step forward from just doing something that’s interesting versus doing something that really will have broader value and make global health progress. Having the conversations at the Annual Meeting is one place, but as you were pointing out, David, we really need to have those conversations throughout the year. I know that’s happened for me in the hallways. Some of the things that I’m most proud of that I’ve worked on started in the hallways of ASTMH and then built from there.

**David Walker:**

That’s really interesting, Julie. I thought I was the only person that ever invented myself, but I encourage you all to do that.

**Dan Bausch:**

Thanks, Julie. I’m smiling inside about Julie because she’s so passionate about what she does. I get to talk with her every week through our ASTMH President leadership calls. There’s no conversation we’ve had over the last two years where she hasn’t mentioned COR NTDs. So, I’m not surprised that it came up here, but that’s great. Chandy, I think you wanted to come in.

**Chandy John:**

This is a great question, and I think, to Gina’s point, there’s a finite amount of time and effort that everybody has. So, you do have to kind of focus your work. I had two wonderful mentors, still have two wonderful mentors, one of whom is right on stage, Jim Kazura, and the other one, Chris King, is out in the audience, and they were role models of doing basic science translational work and clinical trials. You can do them if they’re related to each other. So that’s one of the things I’ve done. But I think that if you’re doing all of those things, which is a lot, and it’s a lot to try to do well, you may not be able to do the implementation piece. You may need to partner with people to do that.

The example I think of is during COVID-19. This was not malaria, which is what I work on, but I was seeing patients with COVID-19. It was very important in children, and it was being neglected in children. And so one had to advocate for children and proper care and prevention measures against COVID-19 in children during the pandemic, but it did get to a point where I thought, “There are other people that can do this better than I can, and if I can rely on them to do it, I can get back to this other work I’m doing in which I have much more experience and expertise.” So, I think we all want to do everything, and you can’t. So, you have to decide what your focus is.

**Dan Bausch:**

Yeah, I was thinking a similar thing. We often all want to do everything. Still, there’s some people who don’t want to do different parts of that and I think that’s fine. Part of it is figuring out what the right team is. Some people are going to focus on generating the evidence in the laboratory or the field, while others are more on the advocacy side. When I’m thinking about younger people who are trying to figure career paths, an important part is just recognizing the right place to go for your training. If you’re primarily interested in advocacy, don’t choose a post-doc somewhere with a supervisor and mentor who’s just really into only the evidence generation, or vice versa.

It’s important to think a little bit of what your profile is now, what you want your profile to be, and make sure that you’re making the right choices to be somewhere where what you want to do is encouraged and where you can get the skill sets to engage in that way.

**David Walker:**

Yes, it is a long journey from an idea to implementation, and I’m probably too old to do it. But the important thing young people can do is to identify what one has enthusiasm for even though one may not know how they would ever get to the end goal. All one has to do is to figure out the next step, and to have in mind what one wants to get out of that next step. Right now, at age 80, my enthusiasm is a vaccine for scrub typhus. We don’t know which antigen stimulates protection. So, we’ve got a long way to go in the laboratory, in preclinical studies, before we’ll ever get to implementation.

But you have to have that goal in mind at the beginning, and you have to be willing to pursue it, and you might get to a point where there’s no company that wants to do it and you have to start a company, and if things work, maybe you can sell your company and go ahead, retire, and watch the implementation stages occur.

**Dan Bausch:**

There you go, key message for the audience there: Go ahead and retire (laughter). Just as I knew that Julie was going to work in COR-NTDs, I knew that David was going to work in scrub typhus! Moving on to the next question, a broader one: I talk with students who are doing their doctoral degrees and going through their training, and there’s a lot of people who are really struggling with that now, feeling it’s really hard to get grant funding, despite putting in a lot of effort. So, there are people saying, “this whole science career thing, is that really going to work out?” I’m sure all of you in your respective roles have thought about this. You’re seeing this concern. What’s your answer to them? How do you keep people enthusiastic and engaged when times seem to be tough?

**James Kazura:**

Earlier this morning, the ex-Presidents had a meeting that addressed ways of improving the experience of students and post-docs attending the Annual Meeting. In previous years, we tried to match a mentor with mentees but the program was abandoned because of lack of participation of sufficient numbers of mentors and mentees. This effort should be reinvigorated in the future. Person-to-person interactions can inspire meaningful discussions that ultimately impact career trajectories of young members of the ASTMH.

**David Walker:**

With online interactions with one another like Zoom and Teams, we can do things now we couldn’t do then.

**Dan Bausch:**

The previous try at developing a mentoring program was before we had easy access to online communications. That was one of the challenges. The interactions often turned out to be kind of a one-off thing; you met the person here at the meeting but after that it was pretty difficult to maintain communication. I remember I was a mentor to a mentee in Australia and we met one time. After that there was no easy way to keep in contact. But now there is the possibility to have that first encounter in-person here at our Annual Meeting and keep things up afterwards online. I think providing mentorship is definitely a role that the Society needs to revisit.

**Figure 3.**
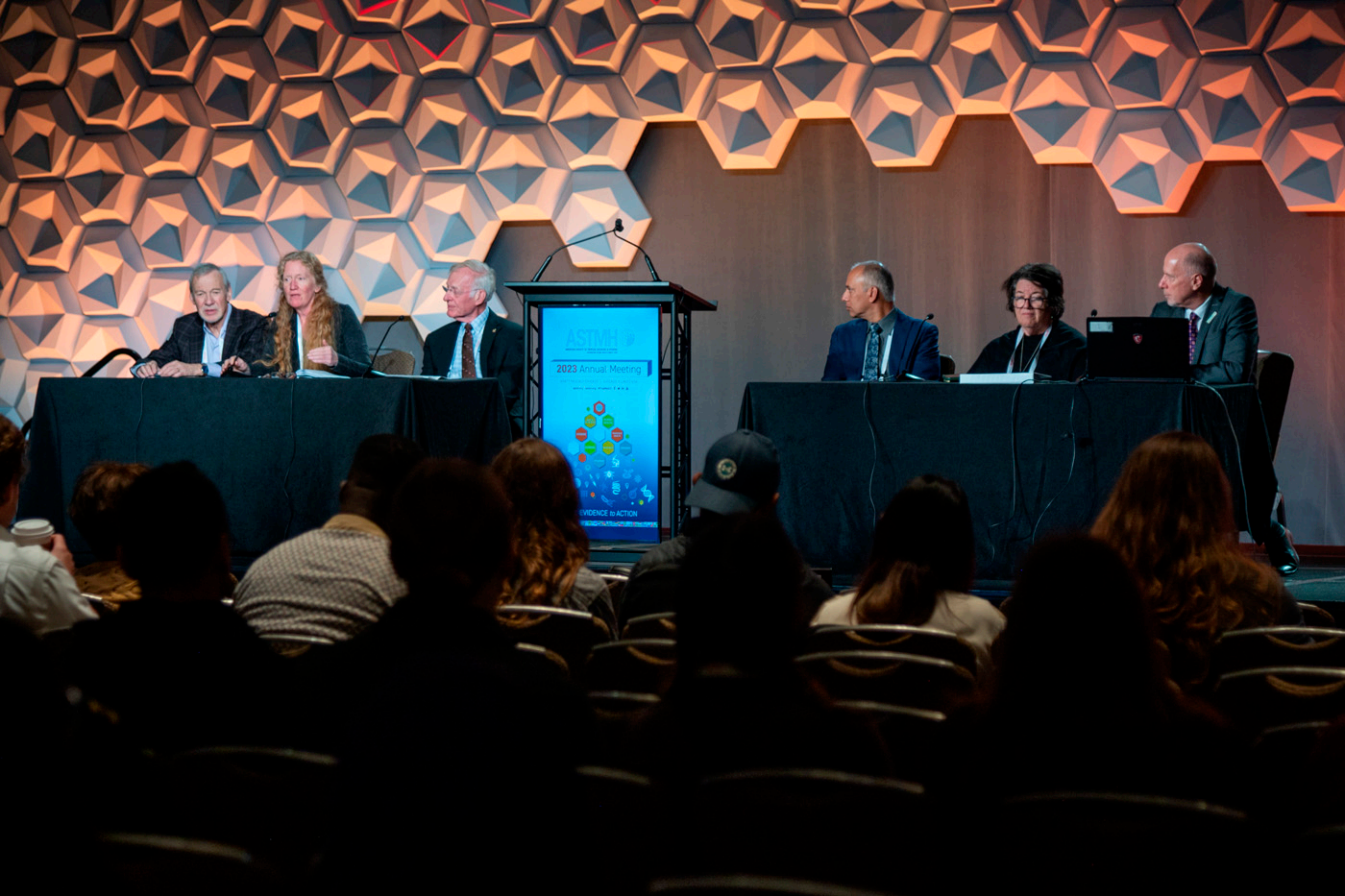
Past-Presidents Panel (from left) Jim Kazura, Julie Jacobson, David Walker, Chandy John, Regina Rabinovich, and Dan Bausch.

**Regina Rabinovich:**

I wonder if we need to do a little work to untangle the word mentorship? Reflecting on those people that have advised me, criticized me, yelled at me over my career, I actually found the yelling very helpful. I have to reflect that one of the people that you would call a mentor, because I was at a deciding point among career paths, is an anthropologist I met very late at night in Cuzco and met randomly two other times in my life when I had a question.

I asked him a question and he came back with some thoughts that were very helpful to me in making a decision or being comfortable with the decision I was making. We all understand what mentorship means in a training program and who is advising you and what you need to do, whether it’s PhD or MD, and the options that you face. But there are opportunities for what I would call mentorship on a lighter strategic level to people who you align with how they think and challenge you. And I would suggest that you seek that variety of voices in your career that I think can be helpful.

**Chandy John:**

So, addressing the question of what do you say in tough funding times, etc., specifically for scientists or physician scientists who are trying to get funding in a tough time, I think most of us would agree that the path to success is less about brilliance and more about perseverance and a very high ability to tolerate rejection, because you get a lot of rejection, and it happens at all levels. It’s happening to all of us now, so don’t think it goes away. So, that sounds discouraging.

And another thing that sounds a little bit discouraging is that there is not a small element of luck. Will you be lucky? Will you be unlucky? But I think the point I would take away from all of this is that everyone who’s sitting up here, their eyes just light up when they talk about what they do and we all say we have the best job in the world. And so if you feel like that for what you’re doing, to the point that it made you forge your own path, you do whatever it takes to keep going.

One can’t guarantee success. But I think that even if you don’t succeed, the effort was worth it because you are doing stuff you love and you think it’s meaningful and important. And honestly, talk to your colleagues and friends; most people aren’t in work that they enjoy, and we are. So, it’s worth striving for.

**Dan Bausch:**

I think that point is true for all of us on stage and, I’m sure, everybody out there in the audience. If you’re in training or relatively younger and you get rejected, do not feel alone. Everybody here has gone through a lot of rejection and in some ways the iteration that comes from that, from writing a grant and then getting it rejected and trying to fix it, is a process that often is helpful in the long run.

The other point, and then we’re going to have to move on from issues that are specific to trainees, and I think I said this the other night in the plenary session, is that tropical medicine people are generally pretty friendly! When you’re in a meeting like this, do not be shy. I encourage students to look around, go to a talk and think “Okay, what is my career path?” Go to varied presentations and explore different profiles. Listen to Gina or Chandy or whoever and say, “Yeah, that’s pretty cool, what that person’s doing. Maybe that’s the kind of profile that I could see myself in.” Then go introduce yourself and talk to them, send them an email, whatever. Most people will be pretty receptive. Don’t sit back. Being assertive with that is important in moving your career forward.

I want to move on now to more general issues about the Society and what our role is. Most people come to a meeting and say,“That’s what the Society is basically—an Annual Meeting.” And of course that’s a major part of what we do, but there’s a lot more that goes on behind the scenes. I don’t mean the operational part—tons of that too—but rather the strategic thinking of what the Society should be. We know that we put on a great meeting every year, but what more can we do to reach out to more people in the world and offer them more opportunity to contribute?

And then of course, not to be a buzzkill, but it all has to be financially viable. Well-meaning people will often suggest that we essentially just provide everything to everybody for free. And it would be great if we could, but if we did that, we wouldn’t be able to continue. Nevertheless, the leadership and Board are giving a lot of thought on how we evolve in this era where there is the possibility of both in-person, online and hybrid meetings, and what the right balance is to accomplish our mission while remaining financially viable.

What should the Society look like 10 years from now? I don’t think there’s ever any question or possibility that we abandon having a great in-person meeting. We all recognize the value of the relationships that we forge and the information that we share in person at these meetings. That’s extremely important. But in addition to that, how should the Society look years from now?

**Regina Rabinovich:**

So, thinking about that really means you’re making projections as to what the world will look like 10, 20 years from now. We know that whether it’s online or in person, it’s an increasingly competitive environment for your interest. So, part of what one does, and part of one’s career is to identify where your home is. For a while for me it was the Academy of Pediatrics, as I was deeply imbued in vaccines as a pediatrician. And then I went and got a one-day pass to something called ASTMH. It was 500 people. And I walked out and said, “I’ve got to come back.”

So, that reflected the evolving changes in my interests. It is also identifying your home base for where you interact with others with similar interests. We need to be very thoughtful about that competitive environment. The Consortium of Universities for Global Health now gathers 4,000 people, the European Infectious Disease Society, Tokyo, Korea, I’m getting invitations for all of these things, but they’re not like ASTMH. Some are meant to be more instructional. They’re not necessarily the scientists presenting, or they are focused on interesting cutting-edge research. So, I think you need to think about what fits best in your career. And for ASTMH, we need to be forward-thinking in terms of who we want to be, in the context of the environment and the changing tools for communication.

**David Walker:**

I have a vision that I think is maybe not very likely, but it is a dream. It could be that 20 years from now, people would come to a room and not sit facing the audience but facing each other, and they would be gathered together around an issue that they really wanted to solve. They would be from all over the world, particularly the part of the world where the disease under discussion was most important. The discussion would be led by people from that part of the world. It would be a time to brainstorm to find the solutions, and then appoint different people in the group to take the leadership on the next steps. That’s pretty wild and probably isn’t very likely, but 20 years from now, who knows?

**Julie Jacobson:**

I think we have tools to build toward that reality now. My team is doing some really interesting work on virtual connections, reaching down across the health system to people that are at the sub-district level virtually, which we thought we would never be able to reach online. These people are now part of the problem-solving and putting in ideas to be tested and tried. I think there’s ways to do this, bringing in virtual modalities, and ways for ASTMH to tap this opportunity more.

There’s been significant discussions around thinking about ASTMH in a regional way. Are there ways in which we can have activities regionally, and we’re experimenting with something on that maybe this year in Africa. So, stay tuned. In the future, I think there is a way to get broader discussion and input and to then have that build up to what happens at the Annual Meeting, where we’re all coming together globally. There really is nothing like being at ASTMH with almost 5,000 people from ∼130 countries this year…130 countries. That’s amazing.

Just think of all the interesting people that are at the meeting sitting next to you. If we capitalize on this as we move into the future, we can structure a way in which to create communications that foster new ideas and new voices being heard. That could be a reality, especially with the tools that we will have in 20 years. Who knows where we’ll be?

**Dan Bausch:**

And I don’t think we’re 20 years away from that, actually.

**Julie Jacobson:**

No, no. Exactly.

**Dan Bausch:**

I think we’re much closer than that. In recent Executive Committee and Board meetings we’ve discussed what are the next steps for us. Again, I don’t think anyone would say we shouldn’t have this wonderful Annual Meeting, but it might be a few years until we have a lot of in-person meetings around the world. That would be much more challenging and costly, entailing a lot of logistical and financial issues to work out. We recognize that many people can’t come to the Annual Meeting for issues of cost or visas or whatever, but there are tools now that could be employed to reach them. Some of the online tools—and no, it’s not the same as in-person, so it’s not one or the other—can supplement what we do now. I don’t think we’re far away from that and have some discussions about trying to put some things together like that in the very near term.

Regarding another outward looking aspect of ASTMH, I often get the question “What role do you see for the Society in the future?” One of the things I would like is that when someone in the general public or in the political/policy sphere has a question about global health or tropical medicine, they automatically think “Well, what does ASTMH say about this?” I don’t think we’re far from that.

I also think that there’s a role for us as a convener of experts. Obviously, we do that in this Annual Meeting, but we could also do it on some specific topics, convening people and meetings in Washington or Geneva or wherever it may be relevant. These sorts of things are in discussion right now and I don’t think we’re that far from at least some baby steps in that direction. Chandy?

**Chandy John:**

One thing that I think we will see even within 10 years, and I’m very excited about it, is more diversity in leadership. So, we have an incredibly diverse Society, and one of the great things about coming to this meeting as opposed to pretty much any other American society that I know is that it’s a very international group. We’re a global Society and I am so excited about Linnie Golightly’s presidency. I’m excited that she is the first African-American President of this Society. I think that’s fantastic, and I think one thing that we all realize from this meeting is that the diversity of the participants is what makes it great.

Getting input from everyone is what makes it great. So, I look forward to seeing people from Africa, Asia, South America being President, being on the Board and helping us lead the Society in new directions, because it will make the Society better.

**Dan Bausch:**

Thanks. I was going to say a similar thing; if you’re out there and interested in contributing, first of all, maintain your membership, but don’t simply leave it at that. There are a lot of other ways to get involved. You can apply to be on the Scientific Program Committee, reviewing the material that comes in and shaping the program. There’s the Young Investigator Award Committee, and quite a few others. So don’t be satisfied to just say, “Okay, I’m a member and that’s it.” You do have to put in your time. If it’s your first year as a member, you might not yet put your hat in for President but get involved and work your way up.

We really want your contribution and for you to build toward leadership roles, especially, but not uniquely, people coming from areas where many of the diseases that we study are endemic. Members from LMICs, we encourage you, in particular, to think about leadership roles in the Society.

**Regina Rabinovich:**

I wanted to make just a plea to the younger, if I may use that phrase, people who are members of the Society: That the answer to what we will be in 10, 20 years is in your hands. What do you need it to be? How is what you need from a society changing over time? And will it reflect what your own changing interests are? And that’s the nature of being a membership group where your voices are the ones that count in terms of where we’re heading.

**Dan Bausch:**

The time’s running too fast, but if you have questions, please step up to the microphone. Introduce yourself, please, and then ask your question.

**Anne McCarthy:**

Thank you very much. My name’s Anne McCarthy. I’m from Ottawa. I was reflecting earlier, I think I’ve been part of this Society for almost 30 years, and one of the big benefits of this Society is how people stay involved for a long time. And as you said, I understand when you were planning this, you asked all the Presidents who would like to get up and present and they all stood up and were happy to do it. So, I would ask, if we can get, there’s a lot of ex-Presidents in the room, is it okay if we get them to stand up so the students can see who they can sort of gravitate through? These people continue to be pillars of the Society, and I think it’s important to recognize.

**Dan Bausch:**

That’s a grand idea. Why don’t we have them all stand up and give them a round of applause? Great, thank you.

**Next speaker (unidentified):**

We call the Society Tropical Medicine. So that’s our team. And the Society might be thinking about when are we going to take this to the tropics? When are we going to take the Annual Meeting to the tropics? It might be an investment, but it’s a good investment. When you look around the room, you see many people from the tropics. And so, if we take it to the tropics, I would bet maybe in 10 years, in five years, even rotating between North America and the tropics every five years or something, that would be a very good investment.

**James Kazura:**

We have had had ASTMH local meetings in the past in overseas sites such as Peru and Kenya. These meetings have been well attended but pose significant financial challenges to the Society.

**Dan Bausch:**

That’s exactly right, and I’ll speak very bluntly. Someone said to me the other day, “Okay, why don’t we have the meeting in…” I think it was Nairobi, Kenya. I replied, “We can have the meeting in Kenya, but you have to guarantee me that we’ll have the same number of people who will register and come to the meeting and pay the same amount because we need to be financially viable.”

I know that’s not how we like to think about things, and that’s not your primary responsibility, but it is the leadership’s responsibility to make sure that we have a viable business model. We definitely *do* want to extend into LMICs. There’s no question about that. But we need to do it in a way that allows us to be financially viable and sustainable. The first forays are probably going to be smaller meetings and online events that have less financial risk. But it’s an important point and it is also the topic of much discussion about how and where to hold future meetings and expand our mission.

To add a bit of background context, as most here know, we had to cancel a couple of meetings during the COVID pandemic—a tough time for everyone to not have those. Since then, we have felt like we needed to stabilize the ship, to have a couple in-person meetings—in Seattle, which we did, a great meeting last year, and another great meeting here in Chicago. Now people are feeling that we’re back in the groove, things are a little bit more stable, and it’s time to think about how we move forward. Meetings overseas, or at least some enhanced engagement overseas, is definitely on the list for discussion.

**David Walker:**

I remember the one was canceled in Puerto Rico. Hurricane hit and it wiped out the place we were going to have the meeting.

**Dan Bausch:**

[Kidding] You do know that’s still USA, right?

**Devy Emperador:**

Hi, my name is Devy. I’m from FIND, in Geneva, Switzerland, and I was thinking a lot about the discussions earlier this morning about the need for students and trainees to look at mentors and to think about mentorship, work with colleagues who are above us, who are in interesting and exciting positions. But I’d also like to argue and kind of push for a lot of our older colleagues who’ve been in this field for a long time, not only to think about being mentors, but also being sponsors.

I think what’s challenging for many younger people is knowing where to go, but also sometimes needing the help to get them to get to where they want to go. It takes a lot of good relationship building between the students or the trainees and the mentors to become a sponsor. But I think it’s quite important to also think of next step for our leaders as well is to champion people to move up.

**Dan Bausch:**

Great. No response, Devy, from the panel, but everybody in the room clapped, including people up here. So, I think we totally agree.

**Marena:**

Marena, Baylor College of Medicine, Honduran by training in tropical medicine. So, for me, ASTMH has been my home, I don’t even know, since when, right? So, I congratulate, certainly this panel, because I think it really amplifies the voices that you represent, which is all of us here. But I want to maybe just have you reflect a little bit more on expanding this concept of bringing more diversity even outside of biomedical or tropical medicine.

I mean, we have wonderful poet laureate communications. Julie, when you were a President, I enjoyed very much all the efforts of bringing the concepts of humility and compassion. Of course, Gina, which I don’t see because she’s over there, bringing more of this connectivity. So can you talk a little bit about bringing other disciplines, like in a panel I was actually chairing, we brought in the legal perspective, the lawyers, bringing engineering, bringing in, of course, the social sciences. Can you speak to how we can help bring all these other voices that we really need? Thank you.

**Dan Bausch:**

I can take a first go at that. Super important. Karen Goraleski, our outgoing CEO, always uses the term “the big tent of ASTMH.” We discuss a lot how big the tent should be, how many people, and what disciplines should be inside it. We have people who’ve been coming to the meeting for 20 or 30 years. When they started, the meeting was really just a small group of scientists talking about their particular pathogen of study, and that was about it. Now it’s grown to have all sorts of different things, and it needs to continue to grow. We discussed this morning with the Past Presidents how, obviously, climate change is an overarching challenge for us. No matter what pathogen or disease you’re interested in, there’s an effect of climate change. And so, we need to bring that into the tent, but many other things as well. As many of you know, I think human rights is an essential principle to be represented in the Society and was the topic of my President’s address last year.

But there are challenges in that the tent is already pretty crowded. Thirteen concurrent sessions throughout the day for almost four days, right? I don’t think anyone wants to go to 15 concurrent sessions. Raise your hand if you’d like 15 concurrent sessions (no hands raised). For most of us this is a great meeting, but after four days you’re like, “Wow, I’m wiped out. I can’t take any more than that.” So, I don’t think we’re going to add on a day or pack in more sessions. So how to expand the big tent? Perhaps with cross-cutting themes? It won’t all be in-person, I think. There are other ways and modalities that we can use and welcome your ideas on this.

One of the big challenges, as well as opportunities, for the Society, is what do we add in addition to an Annual Meeting? What can we do to underscore the value of the Society, and Society membership, throughout the year so that we’re contributing, engaged with each other and the external world, not just one time for four very intense days, but across the year in a manner that is more consistent. And then the other topics that you mentioned, social sciences and climate change, for example. It’s not that they shouldn’t or won’t be represented here, but we need new avenues to have them represented as well. Julie?

**Julie Jacobson:**

I think it comes back to the theme of many voices, and I think this was asked earlier about having your work fit into a solution. That throughput, that is the theme of this year’s meeting, from evidence to action. To do that, you need the anthropologists, you need the policy people, you need people who think and speak very differently to be part of that solution set and problem solving.

I think it would be really interesting to have some conversations at ASTMH about what it takes to have “the big idea”, and to get it actually put at scale into programs. This would help people see that throughput and what it means to see through the idea to implementation. Because the earlier you think about the ultimate endpoint, the more likely it’s going to get there and be more streamlined to get there quickly.

Success comes from all of those different voices being part. You can have something perfect that culturally doesn’t work. There’s lots of really fascinating examples of cultural issues stopping progress, but if you would’ve brought in the right voices to begin with, you could have avoided that pitfall. I think there are ways that we could foster that at the Society, to have that crosstalk and share and honor what everybody’s bringing to the table with their experience.

**Dan Bausch:**

Thanks, Julie. I see that we’re unfortunately close to the end of our time, but I’d like to give everyone a chance to share a last moment or thought. Jim, anything? Last words you’d like to share with the Society and attendees?

**James Kazura:**

Well, to the students, I think we would appreciate your help in implementing efforts to improve mentorship.

**Julie Jacobson:**

We, sitting up here, are not ASTMH. We *all* are ASTMH. This is going to be a co-created future in how we can be most effective as a Society and be most effective in our work. I would like to encourage you all in that active engagement to make it happen.

**David Walker:**

I would like to emphasize that the single most important thing is to identify the thing that stimulates your enthusiasm to pursue it and let it become a passion. Then you really will enjoy your career.

**Chandy John:**

I remember when I came to my first meeting in 1996. It was amazing to me. I thought, “Wow, I have found my people.” It was just incredible. And the thing is, I still feel that way today. And now I meet people who are like me in 1996 and I just am so excited about the passion, enthusiasm that you bring to the Society. So, as Gina said, you’re the future. You’re moving it along, and there are many things we need to do better, but this is a wonderful group to be part of. And maybe the best point to make is people are friendly and approachable here. I was shocked in 1996 to see people, all these people whose names I’d seen on papers, who would answer me when I asked them a question. So, don’t feel shy about doing that. There are amazing people here. Just go up and talk to them.

**Regina Rabinovich:**

To combine the thinking about the future, but also where we grow, I’ve been impressed that sometimes it’s getting people together within the knowledge sets within the Society. An example is I held a meeting of people who work on vector resistance and drug resistance, and it turned out they had never met with each other. And the principles of biology and resistance and the drivers and how to evaluate and the tools you need were very similar. Both groups perceived that their area as very neglected.

So, I think there are ways of thinking not only in bringing other experts from the outside to join in on discussions that we care about – without necessarily bringing in that entire field – but also looking for critical discussions within the Society where we do have the people, but they’ve never had that opportunity to think across rather than up and down.

**Dan Bausch:**

I have nothing to add to all the great comments that were just made, so I won’t say anything other than to thank all the panelists and thank the audience for joining. Enjoy the rest of your day and the rest of the meeting.

